# Molecular epidemiology of *Mycobacterium bovis* in Cameroon

**DOI:** 10.1038/s41598-017-04230-6

**Published:** 2017-07-05

**Authors:** N. F. Egbe, A. Muwonge, L. Ndip, R. F. Kelly, M. Sander, V. Tanya, V. Ngu Ngwa, I. G. Handel, A. Novak, R. Ngandalo, S. Mazeri, K. L. Morgan, A. Asuquo, B. M. de C. Bronsvoort

**Affiliations:** 1 Division of Genetics and Genomics, The Roslin Institute and the Royal (Dick) School of Veterinary Studies, University of Edinburgh, Easter Bush, Midlothian EH25 9RG UK; 2 Microbiology and Parasitology Unit, Faculty of Allied Medical Sciences, University of Calabar, Calabar, Nigeria; 3 Laboratory for Emerging Infectious Diseases, University of Buea, Buea, Cameroon; 4 Tuberculosis Reference Laboratory, Bamenda, P.O. Box 586, Cameroon; 5 Cameroon Academy of Sciences, P.O. Box 1457, Yaoundé, Cameroon; 6 School of Veterinary Medicine and Sciences, University of Ngaoundere, B.P. 454 Ngaoundere, Cameroon; 7 Laboratoire de Recherches Vétérinaires et Zootechniques de Farcha, N’Djaména, Chad; 8 Department of Biomedical Sciences, Faculty of Health Sciences, University of Buea, Buea, Cameroon; 9 Farm Animal Clinical Sciences, School of Veterinary Medicine, University of Glasgow, Glasgow, G61 1QH UK; 10 Institute of Ageing and Chronic Disease, University of Liverpool, Leahurst Campus, Neston, Wirral CH64 7TE UK; 11 Centre for Tropical Livestock Genetics and Health, The Roslin Institute and the Royal (Dick) School of Veterinary Studies, University of Edinburgh, Easter Bush, Midlothian EH25 9RG UK

## Abstract

We describe the largest molecular epidemiological study of Bovine Tuberculosis (bTB) in a sub-Saharan African country with higher spatial resolution providing new insights into bTB. Four hundred and ninety-nine samples were collected for culture from 201 and 179 cattle with and without bTB-like lesions respectively out of 2,346 cattle slaughtered at Bamenda, Ngaoundere, Garoua and Maroua abattoirs between 2012–2013. Two hundred and fifty-five *M. bovis* were isolated, identified and genotyped using deletion analysis, Hain® Genotype MTBC, spoligotyping and MIRU-VNTR. African 1 was the dominant *M. bovis* clonal complex, with 97 unique genotypes including 19 novel spoligotypes representing the highest *M. bovis* genetic diversity observed in Africa to date. SB0944 and SB0953 dominated (63%) the observed spoligotypes. A third of animals with multiple lesions had multiple strain infections. Higher diversity but little evidence of recent transmission of *M. bovis* was more common in Adamawa compared to the North-West Region. The Adamawa was characterised by a high frequency of singletons possibly due to constant additions from an active livestock movement network compared to the North-West Region where a local expansion was more evident. The latter combined with population-based inferences suggest an unstable and stable bTB-endemic status in the North-West and Adamawa Regions respectively.

## Introduction

Bovine tuberculosis (bTB) is caused by *Mycobacterium bovis* which belongs to a group of mycobacteria known as *Mycobacterium tuberculosis* complex (MTBC). The latter includes the following species; *M. tuberculosis*, *M. bovis*, *M*. *bovis BCG*, *M*. *canettii*, *M*. *africanum*, *M*. *pinnipedii*, *M*. *microti*, *M*. *caprae*, the *dassie* and the *oryx* bacillus, and the recently discovered *M*. *mungi* ^1^. These highly successful group of pathogens share an identical 16SrRNA sequence and are up to 99.9% similar at the nucleotide level^1, 2^. This success is reflected in their ability to cause similar pathologies in a wide range of mammalian hosts^3^. For example; some members of MTBC have plagued humanity for millennia evidenced by phthisis and skeletal tuberculosis caused by *M. bovis* and *M. tuberculosis* infections reported from 4000 year old human remains^4, 5^.

The Fulani people who occupy the North-West, Adamawa, North and Extreme North Regions of Cameroon are adept cattle keepers. Their cattle are generally managed in herds of 50–70 animals and grazed in an extensive system with communal unmanaged pastureland^6^. Herds mix with varying number of neighbouring herds at grazing and watering points and have low levels of contact with wildlife such as antelopes and buffalo. Varying numbers of herds take part in a seasonal migration usually grazing areas along river valleys where the contact between herds and also with wildlife are generally higher.

This migratory nature of cattle means that population estimates are highly variable although it is estimated that Cameroon is home to ~5–6 million head of cattle. The largest proportion of this cattle population is found in the Plateau of the Adamawa (~42%), North and the extreme North (~25%) Regions of the country. Trans-boundary cattle migrations are also reported to occur with neighbouring countries such as Nigeria to the west and Chad, Central African Republic to the east^4, 7–11^. In general, there is still limited data on cattle movements within Cameroon although recent work by the same authors revealed the extensive and dynamic nature of the livestock trade network across the country^12^.

There are a myriad of direct social-economic and public health implications of Bovine tuberculosis on a community. This is because the disease not only reduces animal productivity in the later stages but also results in carcass condemnation at slaughter, all of which will impact livestock keeper’s livelihoods^13, 14^.

*M. bovis* is reported to be endemic in Cameroon and has been isolated from meat destined for human consumption^15–17^. There is therefore considerable concern that livestock keepers, abattoir workers and consumers are unknowingly at risk of zoonotic transmission of *M. bovis* (zTB) through contact and consumption of infected animals and their untreated products respectively^18–20^. Currently most countries in Africa, including Cameroon, do not have very robust strategies for controlling bTB and rely predominantly on meat inspection to control the potential public health risk^18, 19^. However, the emerging dairy industry in Africa^13^ and reported higher susceptibility of dairy breeds^21^ to *M. bovis* may increase its zoonotic risk if additional preventive food safety measures are not taken. This is especially relevant because for the first time in the history of World Health Organization’s global TB control strategy, every TB case, regardless of the causative agent matters. Therefore, the zoonotic risks of bTB in sub-Saharan countries like Cameroon, presents a challenge to the World Health Organization’s “END-TB” strategy which is aimed at eliminating human tuberculosis as a public health challenge by 2035^22^.

In this regard, it is critical to understand the spatial dynamics and phylogeography of *M. bovis*, which are largely driven by human behaviour through socio-cultural and economic practices. Such practices include transhumance, communal grazing and livestock trading systems^23^. Since ‘test and slaughter’ is unlikely to be a widely used control tool in low and middle income regions such as in West and Central Africa, spatial patterns, flows and diversity of the pathogen become critical information to understand the epidemiology to develop alternative approaches such as disease free-zoning and/or animal movement restrictions for bTB and zTB control^24^.

Spoligotyping and MIRU-VNTR are still by far the most used typing methods in molecular epidemiological studies of bovine and zoonotic tuberculosis^25, 26^. The latter in combination with deletion analysis has been used to define global clonal complexes of *M. bovis* which include; African 1 (Af1), African 2 (Af2), geographically restricted to Africa and European 1 (Eur1)^27–29^. Af1 is currently restricted to West Africa where it is thought to have originated^27^. It is characterised by the RDAf1 chromosomal deletion, the absence of spacer 30 and clones that are derivatives of SB0944 spoligotype. To date, 95% of Cameroonian *M. bovis* genotypes belong to this clonal complex^16, 17^ with spoligotypes SB0944, SB0953 and SB0955 as the most dominant types reported in the country^16, 17^, with few spoligotypes having been previously reported to be unique in specific areas of the country. MIRU-VNTR typing has not yet been widely used in bTB research in sub-Saharan Africa, there is therefore little epidemiological data linked to genetic diversity at higher resolution that this method would provide^27^.

*M. bovis* is a highly clonal pathogen and diversity is almost exclusively driven by the loss of genetic material which cannot easily be replaced as recombination of chromosomal sequences is extremely rare^9, 27, 30^. Therefore, specific deletions represent useful molecular signatures for any group of *M.bovis* strains. In this regard, we can assume that following the sequential deletions traces the evolutionary chain within and between groups of *M.bovis* strains in a given geographical setting^9, 24, 30^. This attribute is the basis upon which *M. bovis* lineages and clonal complexes have been deduced^28–30^. In the absence of any controls, the accumulation of deletions and hence diversity in genotypes within a given geographical area can be exploited to make inferences on transmission, selection pressure and expansion or shrinkage of the effective population^31–33^. One such molecular based-model that has been used to understand the epidemiology of tuberculosis is the infinite allelic model (IAM) which examines the relationships between mutation (*increase in diversity*) and genetic drift (*reduction in diversity*) under selective neutrality^31, 34, 35^. Under the IAM, a high diversity characterized by a high frequency of singletons suggests a population experiencing a high level of genotype introductions possibly through high level of mutation or re-activation^36^. Furthermore, the variation in diversity could be an indicator of the change in effective population size in an area^31, 36^.

This study describes the molecular diversity and phylogenetic relationships of isolates of *M. bovis* collected from cattle slaughtered in four municipal abattoirs across four administrative Regions of Cameroon with an aim of gaining greater insight into the epidemiology of bovine tuberculosis. This is the most geographically extensive study in a single sub-Saharan country, which has generated both high-resolution molecular epidemiological data but also linked to higher resolution spatial data by including more regional abattoirs and identifying regions of origin.

## Results

### Animal characteristics summary

**Figure 1:**
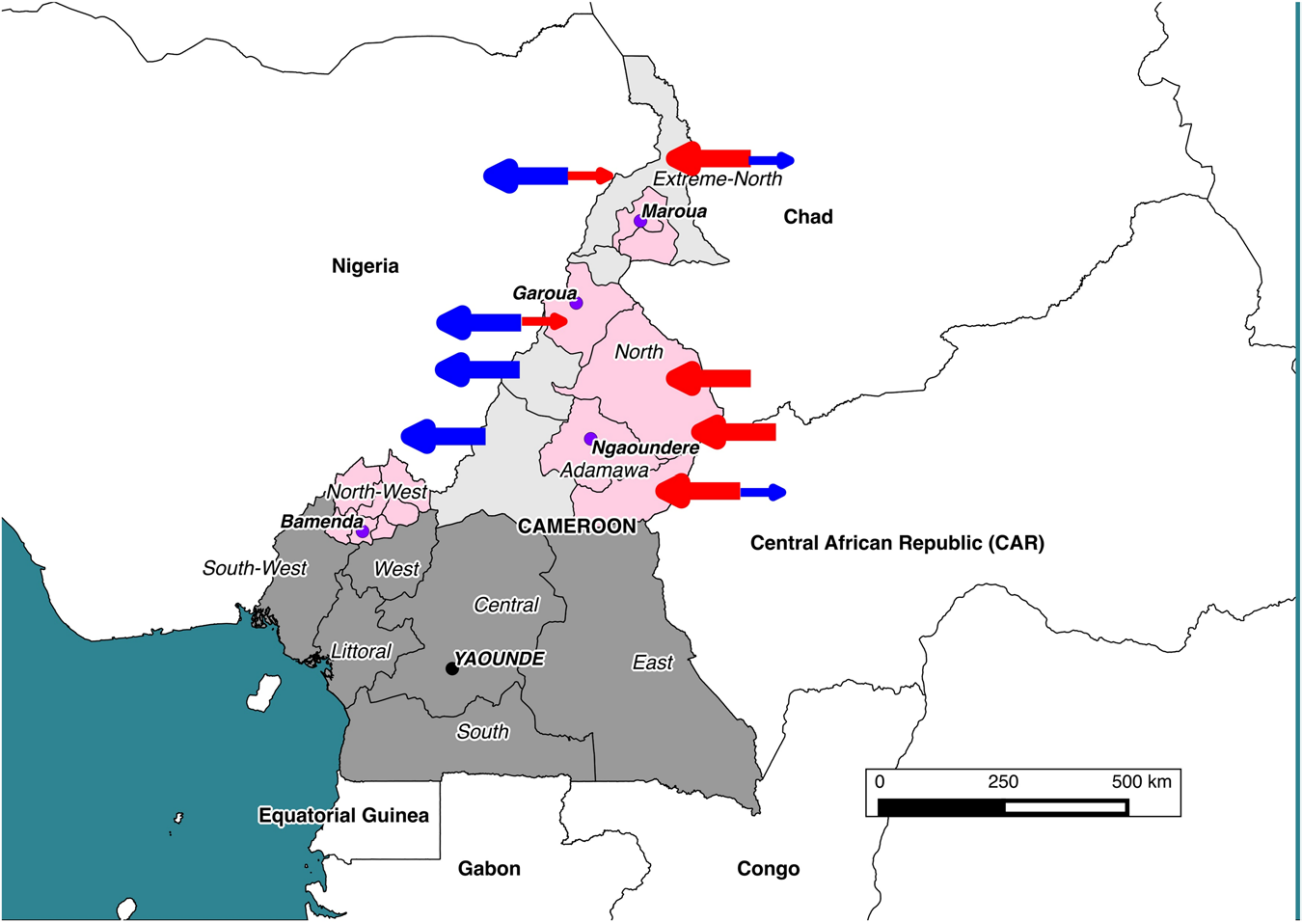
Map showing the location of the 4 municipal abattoirs (purple circles) in the 4 administrative Regions of Cameroon sampled between 2012–1013. The catchments supplying cattle to the abattoirs based on the butchers reporting are also shown (pink shading). Blue arrows indicate general movements out of the country and red arrows movements into Cameroon. The size of these arrows reflects anecdotal views on volumes of animals moved. The figure was generated using QGIS 2.2 (www.qgis.org) and shp files obtained from the GADM database of Global Administrative Areas (www.gadm.org).

From 2,346 examined cattle; 1,129 were at Bamenda abattoir (North-West Region), 935 at Ngaoundere abattoir (Adamawa Region), 122 at Garoua abattoir (North Region) and 160 at Maroua abattoir (Extreme North Region). Figure 1 shows the locations of the abattoirs and the catchment areas sending cattle reported by the butchers. Of these, 207 animals had tuberculosis-like lesions in one or more lymph nodes from which 317 samples were collected for mycobacterial culture (84 animals had more than one sample taken and samples were not collected from 6/207 animals with TB-like lesions due to logistical problems on the day of sampling). A random sample of 182 retropharyngeal lymph nodes was also collected from 179 apparently healthy cattle with no visible bTB-like lesions. (It should be noted that an additional 32 lesioned tissue samples collected during meat inspection could not be linked back to a specific animal. These were thus not included in any parametric analysis but the *M*. *bovis* genotypes from these samples are reported for completeness, as some were novel types). Acid Fast Bacilli (AFB) were observed in 293 of the 499 samples collected, of which 255 (86.7%) were identified as *M. bovis* (Table 1). *M. bovis* was isolated from 150/201 (0.746: 95% confidence interval (CI) 0.682–0.801) animals with bTB-like lesions and 3/179 (0.017: 95% CI 0.006–0.048) without macro lesions.

**Table 1:** Isolation of *Mycobacterium tuberculosis* complex from cattle lymph nodes sampled in four abattoirs in Cameroon between 2012–1013.

Abattoir	Animals sampled for culture	Cultured Lymph nodes	AFB /LN positive animals	*M. bovis* positive animals	*M. bovis* positive lesions
**Lesion (LN)**					
Bamenda	45	69	33/57	31	55
Ngaoundere	106^+^	150	78/120	69	111
Garoua	38	71	35/66	34	64
Maroua	18	27	16/23	16	22
Total	207	317	162/266	150	252
**Non-lesion**					
Bamenda	91	94^*^	20/20	0	0
Ngaoundere	88	88	7/7	3	3
Total	179	182	27/27	3	3
**All Abattoir**	**386** ^**+**^	**499** ^*^	**189/293**	**153**	**255**

^*^This figure includes three animals with lesions from which an extra non lesion lymph node was collected. ^+^It should be noted that samples from six animals here were not cultured. LN & AFB stand for Lymph node and acid fast bodies respectively.

### Diversity and Distribution of *M. bovis* spoligotypes in Cameroon

**Figure 2:**
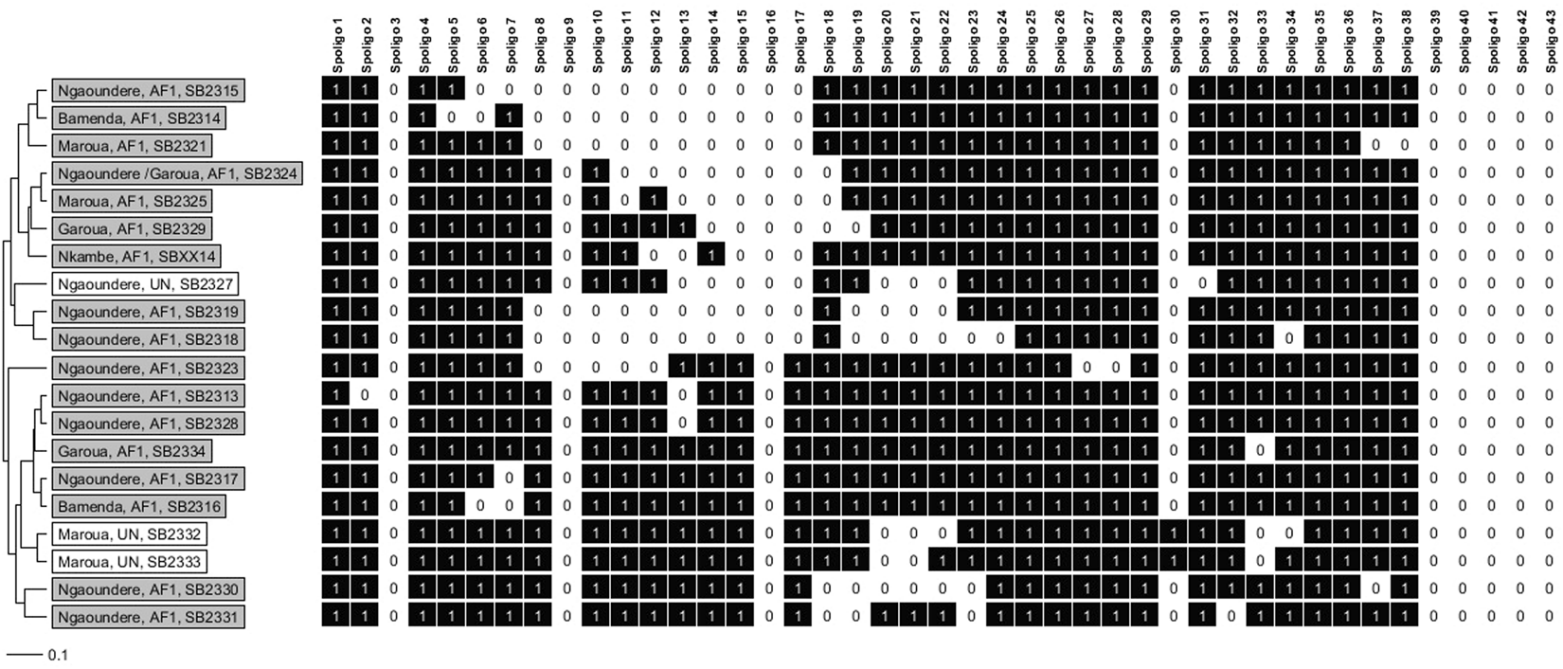
UPGMA phylogenetic tree showing (novel) previously unreported *M.bovis* spoligotypes cultured from cattle from the four abattoirs sampled in Cameroon between 2012-1013. The gray and white shade represents Africa 1 clonal and Unknown complexes respectively. The tree is based on the standard 43 spoligotype spacers.

**Figure 3:**
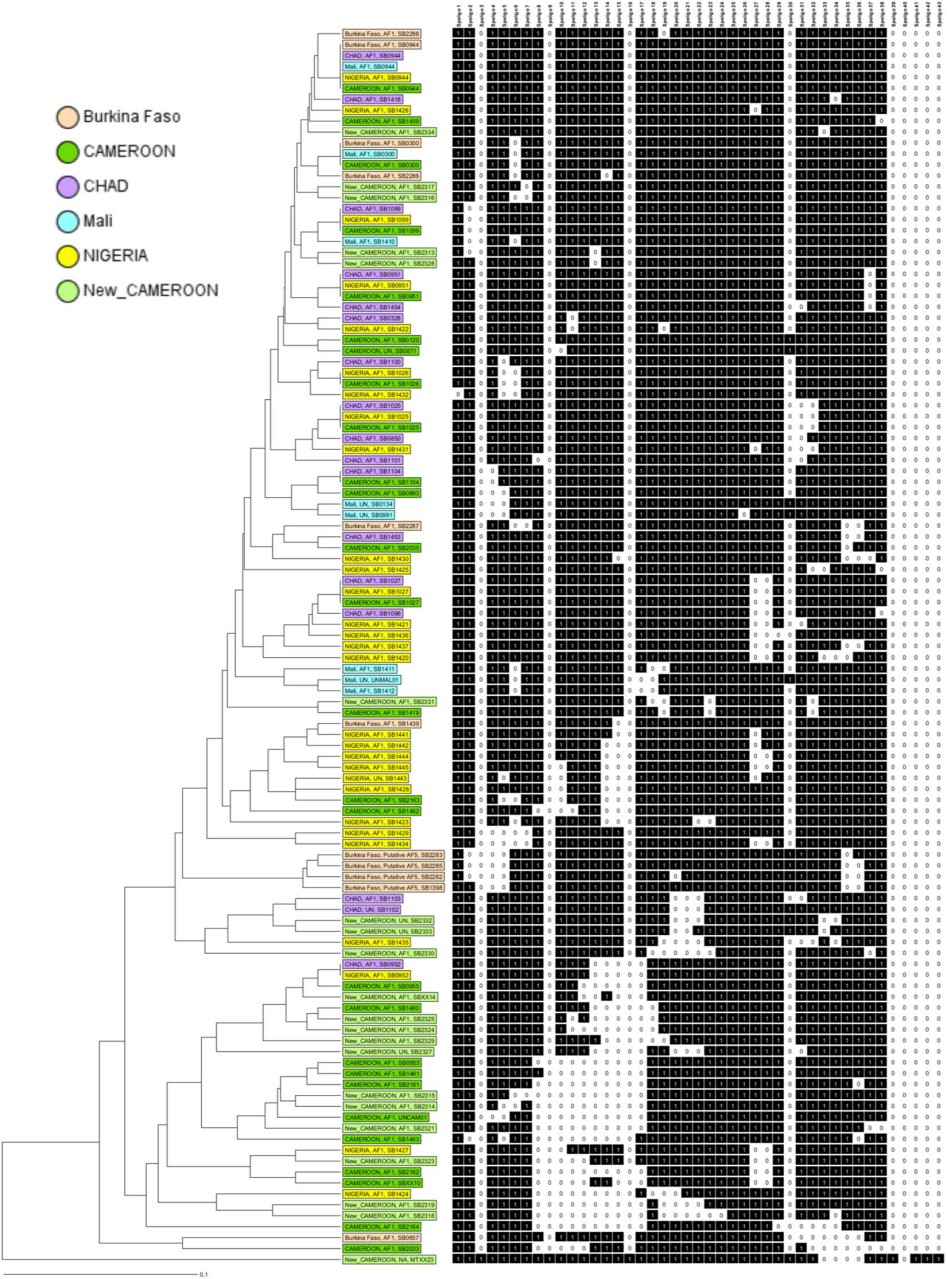
A UPGMA phylogenetic tree showing the unique historic spoligotypes reported in Central and West Africa along with the new previously unreported spoligotypes isolated from cattle from the four abattoirs sampled in Cameroon between 2012-1013. The tree is based on the standard 43 spoligotype spacers.

**Figure 4:**
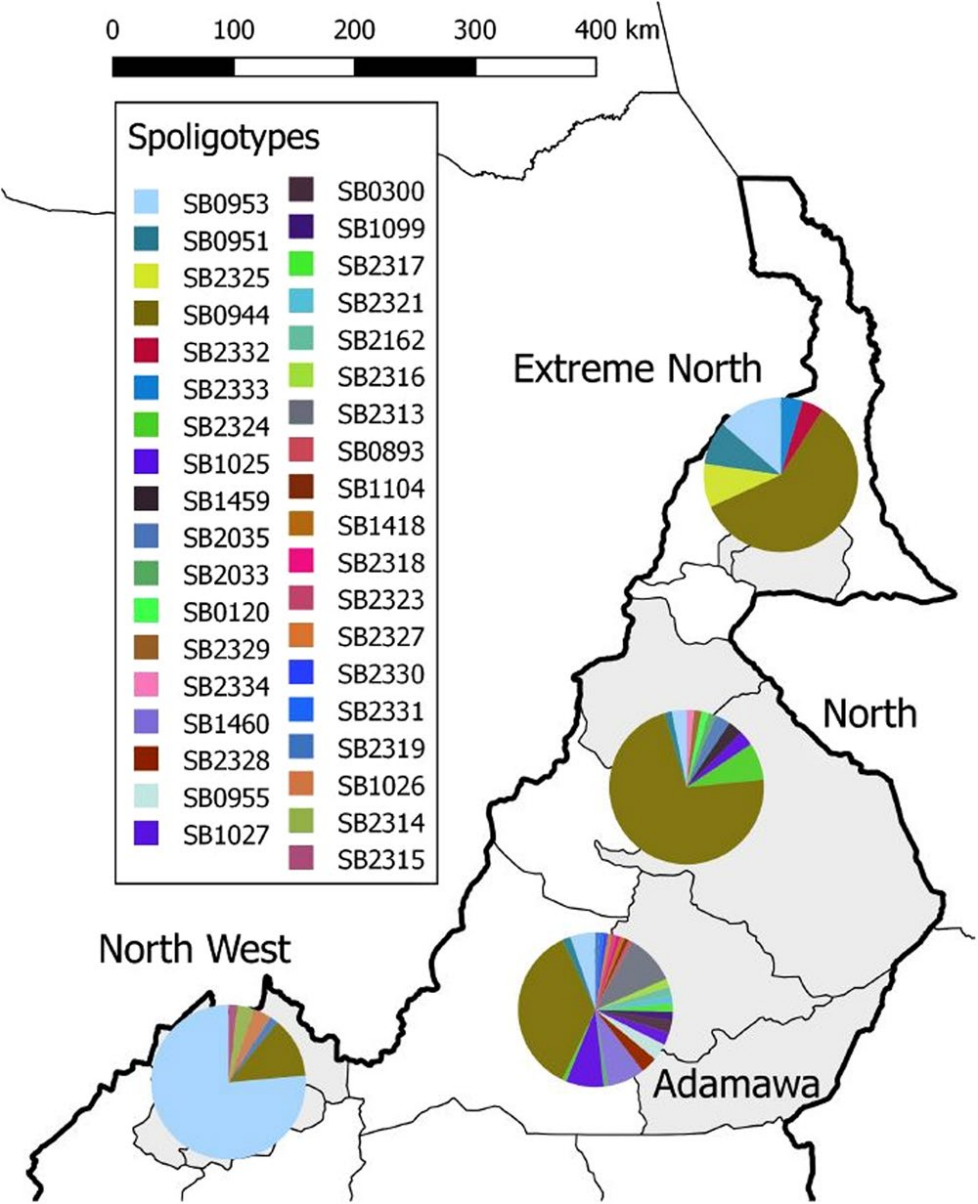
Spatial distribution of spoligotype diversity and dominance in four administrative Regions of Cameroon. This figure was generated using QGIS 2.2 (www.qgis.org) and shp files obtained from the GADM database of Global Administrative Areas (www.gadm.org).

Ninety-eight percent (250/255) of the screened *M. bovis* isolates belonged to the Africa 1 clonal complex. A total of 37 unique spoligotypes were identified whose origin and frequency distribution is shown in Table 2. Comparing the 37 spoligotype patterns to the global database (http://www.mbovis.org), 19 had not been previously recorded. These novel types were submitted to the database and their names were issued (Fig. 2). These include a novel spoligotype SB2320 recovered from one of the 32 cultured samples that could not be linked back to an individual animal (Figs 3 and S1). Absence of spacer 30 was the main characteristic feature of all but SB2332, SB2333 and SB0120 spoligotypes. SB0944 and SB0953 accounted for 42% and 21% of the total number of spoligotypes isolated respectively. It is note worthy that these two spoligotypes were recovered from all the four administrative Regions. SB0944 was predominantly isolated from animals slaughtered in Ngaoundere, Garoua and Maroua accounting for 36%, 72% and 59% of the isolates from these abattoirs respectively (Table 2), while SB0953 predominated in the Bamenda abattoir where it accounted for 76% of observed spoligotypes. Spoligotypes SB1460, SB0955, SB300, SB1027, SB1099, SB1418 and SB0893 were exclusively isolated from cattle slaughtered in Ngaoundere, while SB1026 was exclusively reported from animals slaughtered in Bamenda. Majority of the novel spoligotypes (12/19) were cultured from animals slaughtered in Ngaoundere (Adamawa Region) with the exception of spoligotype SB2324 which was shared by the Ngaoundere and Garoua abattoirs (Table 2 and Fig. 4).

**Table 2:** Distribution of spoligotypes of *M. bovis* recovered from cultured cattle lymph nodes from four abattoirs in Cameroon between 2012–1013.

Spoligo number	Study Site				Overall (n = 255)	Overall percentages	Clonal complex
	Bamenda (n = 55)	Ngaoundere (n = 114)	Garoua (n = 64)	Maroua (n = 22)			
SB0944	7 (3)	41 (27)	46 (26)	13 (10)	107 (66)	42.0	Africa 1
SB0953	42 (24)	6 (4)	2(1)	3(2)	53 (31)	20.8	Africa 1^#^
SB2313*	0	12(7)	0	0	12 (7)	4.7	Africa 1
SB1025	0	9 (6)	2 (2)	0	11 (8)	4.3	Africa 1
SB1460	0	9 (5)	0	0	9 (5)	3.5	Africa 1
SB2324*	0	1 (1)	5 (2)	0	6 (3)	2.4	Africa 1
SB0951	0	2 (2)	1 (1)	2 (2)	5 (5)	2.0	Africa 1
SB0955	0	4 (4)	0	0	4 (4)	1.6	Africa 1
SB2328*	0	4(2)	0	0	4 (2)	1.6	Africa 1
SB0300	0	3 (1)	0	0	3 (1)	1.2	Africa 1
SB1027	0	3 (3)	0	0	3 (3)	1.2	Africa 1
SB2035	1(1)	0	2 (1)	0	3 (2)	1.2	Africa 1
SB1026	2 (2)	0	0	0	2 (2)	0.8	Africa 1
SB1099	0	2 (2)	0	0	2 (2)	0.8	Africa 1
SB1459	0	0	2 (2)	0	2 (2)	0.8	Africa 1
SB2033	0	1 (1)	1 (1)	0	2 (2)	0.8	Africa 1
SB2162	0	2 (1)	0	0	2 (1)	0.8	Africa 1
SB2325*	0	0	0	2 (1)	2 (1)	0.8	Africa 1
SB2314*^#^	2 (1)	0	0	0	2 (1)	0.8	Unclassified
SB2316*	0	2 (1)	0	0	2 (1)	0.8	Africa 1
SB2317*	0	2 (2)	0	0	2 (2)	0.8	Africa 1
SB2321*	0	2 (2)	0	0	2 (2)	0.8	Africa 1
SB0120^+^	0	0	1 (1)	0	1 (1)	0.4	Africa 1
SB0893	0	1 (1)	0	0	1 (1)	0.4	Africa 1
SB1104	0	1 (1)	0	0	1 (1)	0.4	Africa 1
SB1418	0	1 (1)	0	0	1 (1)	0.4	Africa 1
SB2323*	0	1 (1)	0	0	1 (1)	0.4	Africa 1
SB2327*	0	1 (1)	0	0	1 (1)	0.4	Unclassified
SB2329*	0	0	1 (1)	0	1 (1)	0.4	Africa 1
SB2330*	0	1 (1)	0	0	1 (1)	0.4	Africa 1
SB2331*	0	1 (1)	0	0	1 (1)	0.4	Africa 1
SB2332*^+^	0	0	0	1 (1)	1 (1)	0.4	Unclassified
SB2333*^+^	0	0	0	1 (1)	1 (1)	0.4	Unclassified
SB2334*	0	0	1 (1)	0	1 (1)	0.4	Africa 1
SB2315*	1 (1)	0	0	0	1 (1)	0.4	Africa 1
SB2318*	0	1 (1)	0	0	1 (1)	0.4	Africa 1
SB2319*	0	1	0	0	1 (1)	0.4	Africa 1

Spoligotype count per animal by abattoir is given in parentheses. *Novel i.e. previoulsy unreported spoligtypes, ^+^The Africa 1 defining spacer 30 is intact, ^#^This sample gave mixed results therefore could not be conclusively assigned to a clonal complex, Unclassified means that the spoligotype does not belong to any of the currently known clonal complex classifications.

### Genotype diversity of *M. bovis* in Cameroon

Combining spoligo and MIRU-VNTR typing, the 37 unique spoligotypes were further differentiated into 97 unique ‘genotypes’. For example; the predominant spoligotypes SB0944, SB0953 and SB1025 were each further differentiated into 35, 15 and 3 unique genotypes respectively. The rest of the spoligotypes were each subdivided into at most two genotypes (Table S1 and Figure S2).

**Figure 5:**
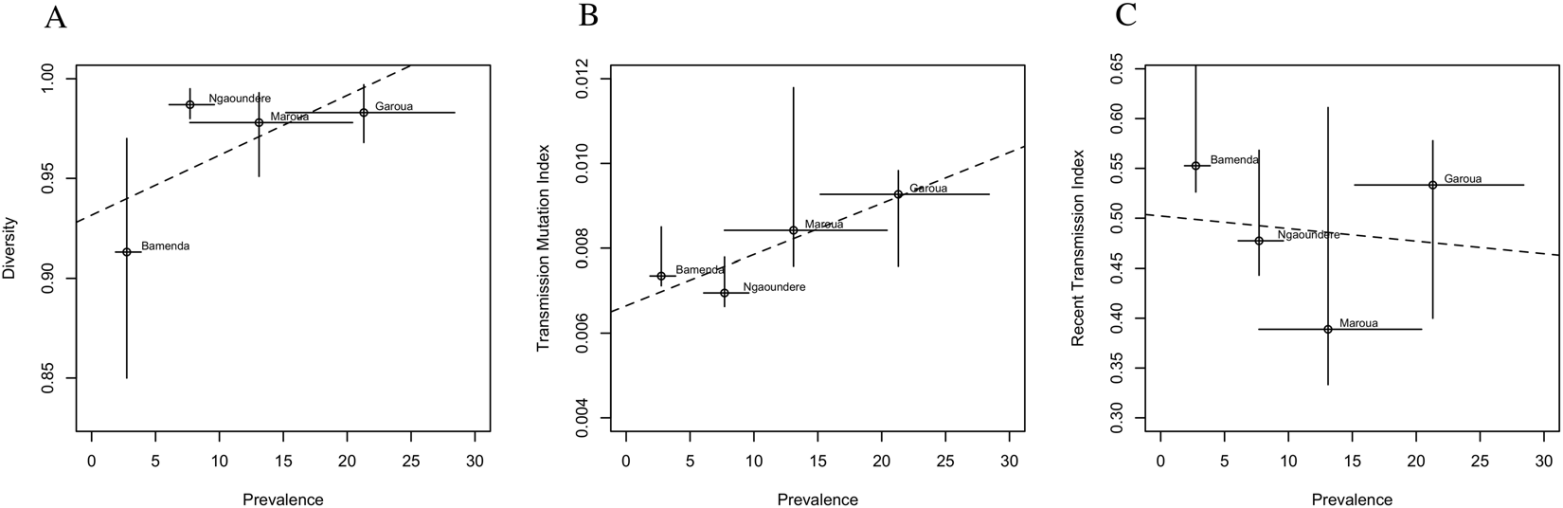
Bivariate plot of the abattoir based prevalence of *M. bovis* and (**A**) diversity, (**B**) the transmission mutation index)- (TMI) and (**C**) the recent transmission index-(RTI in the four abattoirs sampled between 2012–2013 in Cameroon. (B = Bamenda; N = Ngaoundere, G = Garoua, M = Maroua).

Ngaoundere had the highest observed *M. bovis* genotype diversity (0.987) followed by Garoua and Maroua with 0.983 and 0.978 respectively (panel A of Fig. 5 and Table S2). While Bamenda had the lowest *M. bovis* genotype diversity observed at 0.913. It is also noteworthy that ~61% (51/84) of isolates recovered from Ngaoundere abattoir were singletons (Table S2). There was a positive correlation between the genotype diversity and prevalence of bovine tuberculosis for each abattoir (Panel A of Fig. 5). Thirty-four MIRU-VNTR types were shared by more than one spoligotype, demonstrating considerable homoplasticity and reemphasising the need to use the combined spoligotyping and MIRU-VNTR genotyping classification. V2 and V22 accounted for nearly a third of homoplastic MIRU-VNTR types and were mostly encountered in the Bamenda abattoir (Table S3)

**Figure 6:**
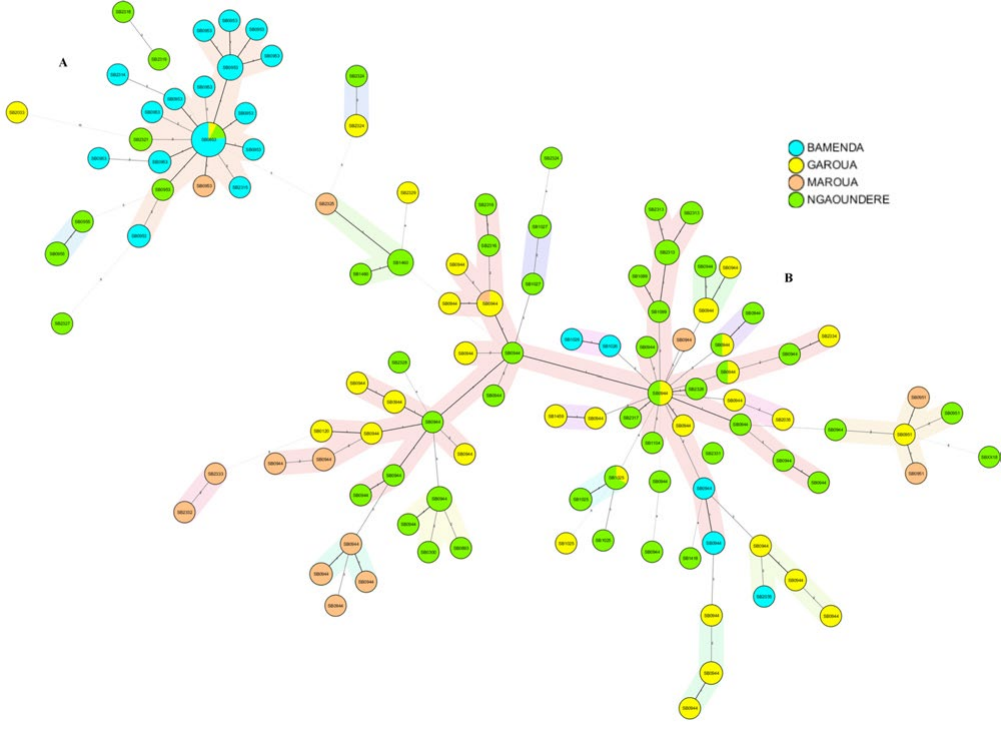
Minimum spanning tree of 97 *M. bovis* “genotypes” based on the combined spoligo and MIRU-VNTR typing of cultured isolates from bTB-like lesions in cattle slaughtered at the four abattoirs sampled in Cameroon between 2012–2013. Nodes are coloured by abattoir of collection and coded with the spoligotype pattern code. The colours light blue, lime green, yellow and brown represent Bamenda, Ngaoundere, Garoua and Maroua respectively.

The phylogenetic relationships between strains are shown in the minimum spanning tree (MST) (Fig. 6). Group A was predominantly recovered in the northern abattoirs (Ngaoundere, Garoua and Maroua), a few of the strains from Bamenda abattoir belong to this group of which SB0944 was the most common spoligotype. Group A can further be sub-divided into a group of genotypes isolated only from Ngaoundere, Garoua and Maroua abattoirs and another that was isolated almost exclusively from Bamenda, Ngaoundere and Garoua. Group B consists of genotypes isolated from Bamenda and dominated by SB0953 and its variants. The predominant genotype in this group was present in three abattoirs whiles its variants were unique to each abattoir (Fig. 6). A between abattoir comparison conducted using Ridom estimated a mean molecular distance between isolates from Bamenda abattoir and the closest sample from the Garoua abattoir to be 1.96 while that between the Bamenda and Maroua Regions was 3.192. On the other hand, the mean molecular distance between isolates from Garoua and the closest isolate from Ngaoundere was 1.596 which is almost half the distance between isolates from Garoua and Maroua.

### Molecular characteristics and cost of typing in Cameroon

**Figure 7:**
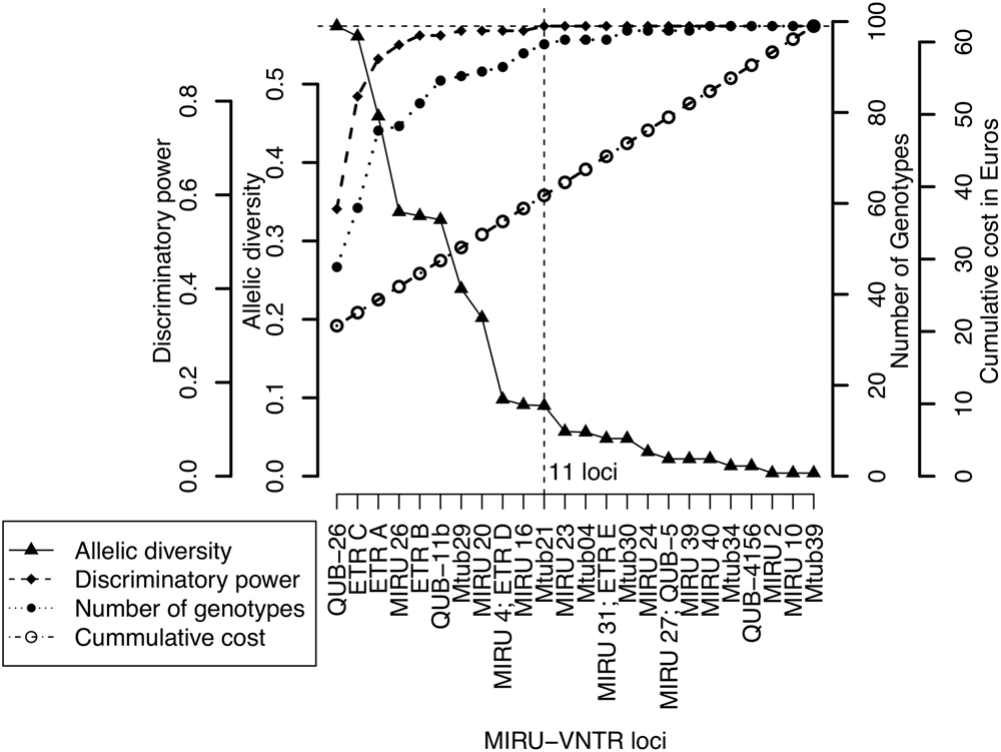
The optimization of cost and benefit (molecular discriminatory power) using number genotypes, discriminatory power, allelic diversity and cost of typing in Euros. The vertical dotted line represents the optimum point at which is the least cost for which one can obtain the highest discriminatory power and number of genotypes. This optimum point is reached with 11 MIRU-VNTR loci used with spoligotyping. This is multi scale two sided Y axis graph, the outer and inner left scales represent the discriminatory power and Allelic diversity respectively while the outer and inner right scales represent the cumulative cost and number of genotypes respectively. The base-line cost of typing is €20 which represents the cost of running only Spoligotyping per sample. The cost increment there after is the cost of adding a MIRU-VNTR loci.

The results of allelic diversity of the 24 loci MIRU-VNTR typing tool used in this study are summarized in Fig. 7 and Table S4. Two of the 24 loci, ETR C, QUB-26, were highly discriminatory (h > 0.55) while four loci, which include ETR A, ETR B, QUB-11b and MIRU-26 were considered moderately discriminatory (0.33 < h < 0.55). The remaining loci had poor discriminatory power with MIRU 2 and MIRU 10 being monomorphic for all the isolates collected in this study. We estimated the spoligotype discriminatory power to be 0.801 (95% CI: 0.759–0.843). This was however increased by 0.188 to 0.982 (95% CI: 0.975–0.989) when spoligotyping was combined with MIRU-VNTR.

Figure 7 also shows the optimization of cost and benefit (molecular discriminatory power) using number genotypes, discriminatory power, allelic diversity and cost of typing in Euros. The cost and number of genotypes detected increases with discriminatory power but the maximum discriminatory power of the combined tool was reached when 11 loci of allelic diversity >0.1 are used which corresponds to an optimum cost of €38.8 per sample. Note that the baseline cost is €19 and is the cost of spoligotyping only, which increases with the addition of each MIRU-VNTR loci.

### *M. bovis* multiple strain infections in Cameroon

Thirty-eight percent (32/84) of the cattle from which at least two samples with lesions were collected contained more than one strain of *M. bovis*. Forty-one percent of these (13/32) were different spoligotypes. The remaining 19 were the same spoligotype but were different MIRU-VNTR types. Ten of these only differed at a single locus. Eighty-four percent (27/32) of multiple strain infections occurred predominantly in adult (dentition score of 4) female, Fulani cattle breeds (white and red Fulani) and most of all were predominantly observed in the Ngaoundere and Garoua abattoir. A quarter (8/32) of the multi-strain infections were detected at the same anatomical site, while the rest (24) occurred in different anatomical sites in the carcass (Table 3). It is note worthy that these 84 cattle from which at least two samples were collected are a proportion of the 207 animals that had TB like lesions in his study, therefore ~40.6% of the animals had lesions in multiple anatomical locations.

**Table 3:** Presence of multiple *M. bovis* strain infections from cattle from four abattoirs in Cameroon sampled between 2012–1013.

Animal ID	Abattoir	Sex	Age (dentition score)	Breed	Different Genotypes (Tissue)	Loci difference
AAA01408^C^	Bamenda	Female	4	White Fulani	SB0953 V99 (Retropharyngeal) SB0953 V94(Bronchial and mediastinal)	2
AAA00722	Bamenda	Female	4	Mixed	SB0953 V99 (Retropharyngeal) SB0953 V54 (supramammary)	1
AAA04513	Bamenda	Female	4	Red Fulani	SB0953 V73 (Retropharyngeal) SB0953 V77 (Retropharyngeal)	1
BBB00503^C^	Ngaoundere	Female	4	White Fulani	SB0944 V72 (Bronchial) SB0944 V75(Mediastinal)	1
BBB00714	Ngaoundere	Female	0	Mixed	SB2324 V107 (Retropharyngeal) SB1027 V107 (Retropharyngeal)	2
BBB00731	Ngaoundere	Female	3	White Fulani	SB0953 V99 (Retropharyngeal) SB0953 V54 (Retropharyngeal)	2
BBB01315	Ngaoundere	Female	4	White Fulani	SB0953 V99 (Retropharyngeal) SB0953 V54 (Retropharyngeal)	2
BBB01421^P^	Ngaoundere	Female	4	Mixed	SB2313 V86 (Bronchial and Mediastinal) SB2313 V79(Bronchial)	1
BBB01428^C^	Ngaoundere	Female	3	White Fulani	SB2319 V117 (Mediastinal) SB2318 V117 (Bronchial)	0
BBB01635	Ngaoundere	Female	4	White Fulani	SB0944 V39 (Mediastinal) SB0944 V42 (Mediastinal)	1
AAA01702^C^	Bamenda	Female	4	Red Fulani	SB0953 V77 (Retropharyngeal) SB0953 V99 (Mandibular and Prescapular)	1
BBB01533^C^	Ngaoundere	Female	3	White Fulani	SB0944 V25 (Mediastinal) SB0944 V21 (Bronchial)	1
BBB01723^C^	Ngaoundere	Female	4	Red Fulani	SB0944 V48 (Retropharyngeal) SB1025 V50 (Mediastinal)	5
BBB01870^C^	Ngaoundere	Female	4	Mixed	SB0944 V42 (Mediastinal) SB0944 V38 (Bronchial) SB0944 V60 (Lungs)	6
CCC00107^C^	Garoua	Female	4	Red Fulani	SB0944 V29 (Bronchial) SB0944 V75 (Mediastinal) SB0944 V34 (Lungs)	4
CCC00320^p^	Garoua	Male	4	White Fulani	SB0944 V18 (Bronchial) SB0944 V30 (Lungs)	1
CCC00419^C^	Garoua	Female	4	Red Fulani	SB2035 V8 (Retropharyngeal) SB0944 V6 (Mediastinal)	1
CCC00509^C^	Garoua	Female	5	White Fulani	SB0944 V63 (Retropharyngeal) SB0944 V64 (Bronchial) SB0944 V65 (Lungs)	3
CCC00329^C^	Garoua	Female	4	White Fulani	SB0944 V12 (Mediastinal) SB1459 V12 (Lungs)	0
CCC00502^C^	Garoua	Female	4	Red Fulani	SB0120 V71 (Mediastinal) SB0944 V71 (Lungs)	0
AAA02506^C^	Bamenda	Male	4	White Fulani	SB0953 V106 (bronchial and mediastinal) SB2035 67 (bronchial)	4
AAA04604^p^	Bamenda	Male	2	Red Fulani	SB0953 V99 (bronchial) SB0953 V77 (bronchial)	1
BBB01725^C^	Ngaoundere	Female	4	Red Fulani	SB0944 V104 (retropharyngeal) SB1025 V50 (Liver)	5
BBB01808^C^	Ngaoundere	Female	4	White Fulani	SB0944 V99 (lungs) SB2328 V5 (bronchial & mediastinal)	4
BBB01835^C^	Ngaoundere	Female	4	White Fulani	SB0944 V99 (Liver) SB0944 V89 (retropharyngeal)	1
BBB01919^C^	Ngaoundere	Female	4	Mixed breed	SB0953 V68 (retropharyngeal) SB1460 V92 (bronchial)	2
CCC00119^C^	Garoua	Female	4	Red Fulani	SB0944 V99 (bronchial) SB0944 V34 (lungs & Liver)	2
CCC00219^C^	Garoua	Female	4	White Fulani	SB0944 V34 (mandibular) SB0944 V99 (lungs)	3
CCC00303^C^	Garoua	Female	4	Red Fulani	SB0944 V1 (retropharyngeal) SB1459 V12 (bronchial)	5
CCC00429^C^	Garoua	Female	4	Red Fulani	SB0944 V91 (retropharyngeal) SB2033 V92 (bronchial)	1
DDD00321^p^	Maroua	Female	4	Red Fulani	SB2332 V11 (bronchial) SB2333 V11 (Lungs)	0
DDD00403^C^	Maroua	Female	5	White Fulani	SB0944 V19 (retropharyngeal) SB0944 V45 (bronchial) SB0944 V46 (Lungs)	7

^P^Pulmonary mixed infection and ^C^Compartmentalized mixed infection.

### Estimating *M. bovis* transmission in Cameroon

In order to characterize *M. bovis* transmission using genotypic information, parameters like the most recent transmission index (RTI) and the transmission mutation index (TMI) were used. These parameters were estimated from empirical molecular data using formulae 3 & 4 (see Table S2) i.e.; g = 17, n = 37 and v_i_ = 11 for Bamenda, g = 26, n = 42 and v_i_ = 9 for Garoua, g = 11, n = 17 and v_i_ = 3 for Maroua and g = 46, n = 84 and v_i_ = 24 for Ngaoundere respectively. The estimates were then plotted against the underlying *M. bovis* prevalence estimates at each abattoir (Fig. 5). Here positive and negative correlation between TMI, RTI and prevalence respectively was observed (Panel B&C of Fig. 5). However, the within-Region recent transmission or contact frequency was highest and lowest in North-West (Bamenda) and Adamawa (Ngaoundere) Regions respectively (Figure S3). The highest frequency of contacts between Regions i.e. the North-West and the rest of the Regions appears to be primarily driven by variants of SB0953 (Figure S3).

**Figure 8:**
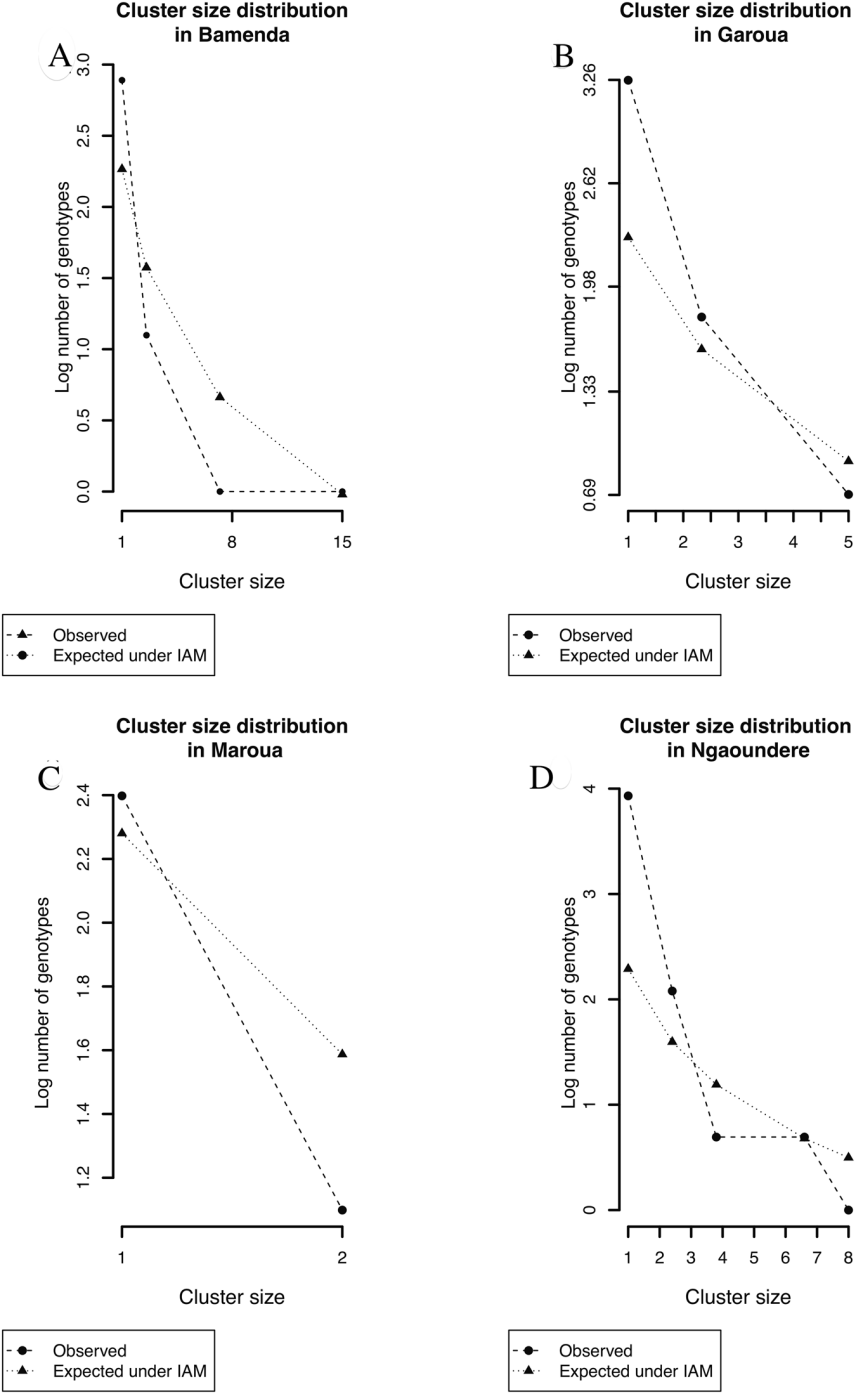
The distribution of the observed genotype cluster sizes (i.e. the number of times a particular genotype occurs at an abattoir) compared to what would be expected under the infinite allelic model (IAM) in the four Regional abattoirs in Cameroon. (A = Bamenda; B = Garoua; C = Maroua; D = Ngaoundere).

In addition, population based dynamics of bovine tuberculosis were explored by fitting our data to the theoretical Infinite Allelic Model (IAM). The model fits, i.e. the observed and expected distribution of genotype cluster sizes for each abattoir, are shown in Fig. 8. The typical IAM distribution of high frequency of genotypes with clusters of size 1 (i.e. singletons) which decreases with cluster size was observed in this study. Specifically, Ngaoundere had the highest proportion of singletons, 61% (51/84), compared to Bamenda which had the lowest, 40% (15/37) (Table S2). There was no statistical difference between the observed and expected genotype cluster size distributions in Bamenda (p = 0.28), Ngaoundere (p = 0.26), Garoua (p = 0.21), or Maroua (p = 0.15) abattoirs respectively, so our data fits the IAM model. The IAM model and its assumptions therefore could be used to explain the epidemiological dynamics of bovine tuberculosis in the four Regions of Cameroon. It should however be noted that transmission and cluster size characteristics were calculated based on *M. bovis* strains recovered from *M. bovis* culture positive animals from which only one genotype was recovered (Tables 1 and 3).

## Discussion

This study reports the molecular diversity and phylogenetic relationships of *M. bovis* isolated from cattle slaughtered in four administrative Regions. Furthermore, we quantitatively exploit the molecular relationships to improve epidemiological understanding of bovine tuberculosis in these four Regions of Cameroon.

The use of population-based molecular markers of infectious pathogens is critical in developing targeted control strategies, which are much needed in resource-limited settings of the world. Such methods exploit the unique host population-driven configurations of pathogen genotype clustering, cluster-size distribution, diversity and spatial distribution to infer contact activity, potential transmission events^31, 37^ and most importantly to identify hotspot of disease.

### *M. bovis* clonal complexes in Cameroon

The vast majority of *M. bovis* isolates from Cameroon belonged to the Af1 clonal complex. The distribution and frequency of spoligotypes appear to be similar to those reported in Chad and Nigeria^27^. Previous studies have alluded to this similarity in molecular profiles and suggested Cameroon as the source of the Af1 complex, which has so far been reported as far west as Mali^27^. Interestingly, the physical distance between Mali and Cameroon is also reflected in the increased genetic distance between strains from these two countries^27^ (Table S5). If indeed this is the case, then the Sahel and West African transhumance cattle movement^38^ might be the driving force responsible for gradually spreading these *M. bovis* clonal variants westwards (Fig. 1).

We observed a small proportion of strains with a spoligopattern with spacer 30 missing which did not belong to the Af1 clonal complex or any of the other defined clonal complexes to date^27, 28^. One of these was interestingly a SB0120 strain with an Af1 chromosomal deletion. This is the first report of SB0120 with an Af1 chromosomal deletion, the details and implication of which will be explored at a later date. In general, the distribution and population structure of *M. bovis* clonal complexes superficially appears to have remained consistent with earlier reports^16, 17, 39^, but this fails to capture the details revealed by higher resolution analysis.

### Diversity of *M. bovis* in Cameroon

Thirty-seven unique spoligotypes were recovered in this study, 19 of which were novel. The most prevalent spoligotypes were SB0944 and SB0953 which is consistent with previous studies in Cameroon^16, 17, 39^. Interestingly, although a recent study revealed that SB0944 was the most predominant spoligotype, the second most predominant spoligotype was SB0955^16^ and not SB0953 as in the current study. In fact SB0955 was not isolated in the current study even when it had been exclusively reported in the Adamawa Region^16^. It is worth noting that majority of the novel spoligotypes (12/19) were recovered from the Adamawa Region possibly underscoring its central role in the epidemiology of bovine tuberculosis in Cameroon^16^.

This is not only the largest *M. bovis* spoligotype diversity reported in West Africa; Nigeria (28), Chad (17) and Mali (7) but Africa at large^27^. One could argue that the number of unique spoligotypes discovered in an area reflects the general interest and frequency of sampling, however, there has been more sampling for *M. bovis* infections in Ethiopia and South Africa yet these countries still fall behind the number of unique spoligotypes currently observed in Cameroon^28^. At a within Cameroon level, the highest and lowest diversity based on the combined typing tool was observed in the Adamawa (Ngaoundere abattoir) and the North-West (Bamenda abattoir) Regions respectively. The drivers for this difference in diversity are likely linked to animal movements within and between countries. In Cameroon, the major route of movement is from the northern Regions to the densely populated areas of the south^16^, while between country cattle movements occur east to west, from Central African Republic and Chad through Cameroon (mainly the North and Extreme North) to Nigeria, driven by the animal protein demands from its ~200 million inhabitants^38, 40^ (Figs 1 and S1).

### Phylogenetic relationships of *M. bovis* in Cameroon

In general phylogenetic clustering of a pathogen’s genotypes can be linked to spatial and temporal characteristics of hosts in outbreak and endemic settings^24, 41, 42^. In this study, *M. bovis* strains cluster in two broad geographical groups (A&B). Group A centred on spoligopattern SB0944 is predominantly present in the 3 northern Regions represented by Ngaoundere, Garoua and Maroua, although a few unique (not shared) genotypes were also isolated from cattle in Bamenda abattoir. All the shared identical genotypes in group A which represent recent contacts, were either from Ngaoundere and Garoua or Garoua and Maroua. This inferred connectivity between the North, Adamawa and Extreme North Regions seems plausible given the physical distance between them and could either indicate shared drivers of bovine tuberculosis or simply a shared source of animals slaughtered in these three abattoirs (Fig. 1). The other major grouping, B, centred on SB0953 is predominantly present in the North-West Region as recovered from Bamenda abattoir. This dominant spoligotype was also recovered in cattle slaughtered in Ngaoundere and Garoua abattoirs. This wide spatial distribution of closely related variants of SB0953 indicates a shared and maintained ancestry and or more recent host contacts/movement of infected cattle between the North-West, North and Adamawa Regions.

The phylogenetic relationships also suggest that there are more within Region (North-West) cattle interactions than between Regions (Figure S3). This observation is analogous to the argument made by Muller *et al*.^27^ 2009 that country specific genotypes are discernible because the between country animal contact is not frequent enough to mask the local epidemiology. Therefore, the typical geographical clustering of genotypes observed in the North-West is discernible because cattle contacts/interactions between the North-West and other Regions are limited to a level that cannot mask the local epidemiology. However, the same does not appear to be true for the Adamawa, North and Extreme North Regions where significant overlap of genotypes is observed. This also may be because sampling was at the large municipal abattoirs so it is possible that Ngaoundere and Garoua receive cattle from the same administrative Divisions (Fig. 1).

### The molecular epidemiology of *M. bovis* in Cameroon

In tuberculosis epidemiology, spatial clustering of identical or closely related genotypes is considered an indication of recent contact or transmissions within a population in a specific area^31, 33, 34, 43–45^. In Cameroon, we observed that Bamenda had the highest frequency of genotype pairs suggestive of recent host contact, which is also reflected by the recent transmission index (RTI). On the other hand, the lowest frequency of identical genotypes was observed within the Adamawa Region. Furthermore, on average less than 1% (0.74) of the transmission are predicted to have generated new *M. bovis* genotypes across the four Regions. The high frequency of recent transmission and or animal contacts, limited evidence of locally generated new genotypes (low TMI), coupled with low diversity and disease prevalence suggests an unstable, highly dynamic and or increasing disease status in the North-West Region (Fig. 1). By comparison, although the Adamawa Region had the lowest TMI, approximately 61% of all the genotypes encountered in the Adamawa were singletons therefore it could be hypothesised that these unique genotypes are introductions from other sources possibly through animal movements or migrations^16, 38^.

Multiple strain infections in human tuberculosis (i.e. genetic diversity within a host) are not only an indication of high transmission pressure at a population level but also give insights into the host-pathogen relationship at the individual level^46^. Host-pathogen relationships have been inferred from strictly compartmentalized mixed infections where different genotypes/strains are found in different anatomical compartments while high transmission pressure has been linked to pulmonary multiple strain infections^47^. In this study 15.5% (32/207) of slaughtered cattle had multiple strain/genotype infections, the majority of which were observed in older female cattle in Ngaoundere and Garoua abattoirs (Table 3). We did not observed any associations of statistical significance between the age, the sex or the breed of an animal and whether the animal had single or multiple strain infections. The apparent predominance in older females reflects the bias in the population passing through these abattoirs^15^. However, sex, age and breed of animals will be fully explored in our subsequent analyses and publication based on next generation sequence markers. Interestingly, multiple strain infections have also been reported in Ethiopian cattle, where in the worst case, up to six different strains were observed in a carcass^47^. In human TB, multiple strain infections have been used to identify “social pockets”, a term coined for individuals who once infected are likely to have multiple exposures by virtue of their social network^48^. Based on this notion, it is more likely that these multiple strain infections are indicative of “social pockets” rather than high transmission pressure especially in the Adamawa, specifically animals originating from the administrative divisions of Mayo Rey and Benoue suggests that these divisions may be home to these “social pockets”^46^.

Features of genetic cluster size distribution have also been used to quantitatively understand population dynamics of tuberculosis using the infinite allelic model (IAM)^31, 36^. According to Luciani^31^ under the IAM a host population that has a high pathogen diversity usually characterized by a high frequency of singletons (unique genotypes) is suggestive of a population experiencing constant addition of exogenous unique genotypes or a high rate of reactivations^31^. Based on the observations in this study, the isolates from Ngaoundere abattoir seems to typify such a population (Fig. 8). Therefore, it could be hypothesized that the high frequency of singletons observed in Ngaoundere is more likely due to constant additions rather than an inherent generation of new genotypes due to mutation, especially given that only 0.74% of transmission events are predicted to generate new genotypes. The constant addition explanation is plausible given the cyclic Sahel-West African transhumance (SWAT) movements^40^ to which the Adamawa Region is central (Fig. 1). The majority of the documented transhumance routes within the SWAT^40^ go through these northern Regions moving livestock from as far east and west as Chad, Central African Republic and Mali respectively. This could explain the similarity of spoligotype profiles between Mali, Chad^27^ and the Adamawa Region and why the North-West Region, which is geographically more isolated has very different genotypes and relatedness patterns (Table S5 and Fig. 1).

Finally, genetic diversity is directly proportional to the effective population size^31^, therefore, in this regard, Ngaoundere and Bamenda representing the Adamawa and North-West Region would have the highest and lowest effective population size respectively. This however seems counter-intuitive to Bamenda given the high frequency of recent transmissions, unless this is an indication of a more endemically unstable bovine tuberculosis epidemiology in the region. Indeed, such complexities are expected for a predominantly airborne disease whose indices like RTI and TMI are more than likely influenced by a multitude of factors the granular nature and scale of which will not be explored in this article. However, it is noteworthy that since our data fits the theoretical IAM, our findings therefore suggest that the effective population size within the different Regions of Cameroon is not expanding. So the unstable and stable characteristics observed in the North-West and Adamawa Regions are likely due to factors peculiar to these Regions. Such population-based bTB insights have a specific and timely resonance in these countries because of their rapidly developing and expand dairy production systems^13^. This comes at a time when the human population is also significantly increasing in these countries, which means now more than ever intensification is the most viable livestock management option. This type of management is however predicted to increase the prevalence of bovine tuberculosis given that it is a “crowding disease”^21^. Therefore, identifying disease drivers and hotspots using such robust molecular technics offers the best chance for designing cost effective and public health-centred strategies for dairy development and expansion in Africa.

### Molecular characteristics and cost of typing in similar settings

Consistent with our findings MIRU-VNTR typing is widely reported to be more discriminatory than spoligotyping^49^, an attribute that is a cumulative function of each individual loci’s allelic diversity^50^ more than likely fine-tuned by factors specific to a geographical area^47^. ETR C and QUB-26 were the most discriminatory MIRU-VNTR loci in agreement with reports from Burkina Faso^51^, Nigeria^52^, Chad^27^. However, the allelic diversity of ETR C was three times higher than that reported in Burkina Faso emphasizing the variation in allelic diversity and utility in different geographical settings with different strains. The three loci that exhibited the lowest allelic diversity in this study MIRU 2, MIRU10, and MIRU 39, have also been reported to have low allelic diversity elsewhere in West Africa^27, 51, 52^.

Identifying combinations of typing tools that can provide the optimal granularity required to answer molecular epidemiological questions is not trivial especially in resource limited settings like Cameroon. We have therefore analysed the relationship between the number of genotypes, discriminatory power and allelic diversity to help in this regard. Our analysis shows that with a combined typing tool using the spoligotyping and 11 of the most diverse MIRU-VNTR loci most of the genotypes can be identified.

In economic terms, the analysis identified an optimum cost of €38.80 per sample compared to €62.20 used for this study where 24-loci were used. So although this optimum point has the potential of missing a few genotypes, it saves nearly half the cost of typing *M. bovis*. We strongly recommended that MIRU-VNTR is not used in isolation given its high potential of homoplasy as demonstrated in this study (Tables S1 and S3). We also recognise that the current use of whole genome sequencing (WGS) in *M. bovis* epidemiology is expected to eclipse the use of spoligotyping and MIRU-VNTR typing globally. This is, however, likely to take significantly longer in Africa because of the following; (a) the vast majority of comparable digital molecular data that exists today in Africa is based on spoligotyping and MIRU-VNTR, (b) Although the cost of WGS is rapidly coming down, it is still cheaper, less data intensive and robust enough to use spoligotyping and MIRU-VNTR typing. These factors taken together suggest that our cost versus benefit (molecular discriminatory power) optimization will be useful for most laboratories in Africa as they transition to WGS.

Due to security reasons, we could not collect as many samples in the North (Garoua) and the Extreme North (Maroua) Regions as we did in the North-West (Bamenda) and the Adamawa (Ngaoundere) Regions. We recognize that this inherently has the potential of limiting the analytical power for the North and the Extreme North Regions and the diversity reported from these Regions. However, although this difference in sample size may affect the total diversity observed, the comparisons of transmission and diversity parameters between Regions account for the different sample sizes. But in order to not over interpret these smaller samples, our conclusions on transmission and population dynamics have been limited to the North-West and Adamawa Regions.

## Conclusions

This is the largest and most comprehensive study on *M.bovis* molecular epidemiology in sub-Saharan Africa. Cameroon is often described as Africa in miniature due to its huge geographical and cultural diversity. It now can also claim the highest *M. bovis* spoligotype diversity on the continent so far reported. Furthermore, we have demonstrated that by trying to understand the different patterns of diversity in the different administrative Regions of Cameroon we can begin to understand their different epidemiology.

The lower prevalence, diversity and TMI coupled with evidence of higher recent transmission in the North-West compared to the three other northern Regions is potentially an indication of an unstable endemic status for bovine tuberculosis in the North-West Region with a recent local expansion/epidemic. By comparison, the northern Regions appear to have a more stable state possibly driven by higher levels of cattle mixing and cross border animal movements. This is also supported by the genotypes prevalent in these Regions, with the North-West dominated by a much more recent strain, SB0953, while the northern Regions are dominated by the ancestral pattern of SB0944. The molecular and epidemiological findings in this study all point towards an active livestock movement especially in the Adamawa and North Regions. Detailed investigation of animal movements in Cameroon is in progress and will be linked to the molecular epidemiology of *M*. *bovis* in Cameroon.

Taken together, the work demonstrates the utility of granular molecular and spatial data in understand the epidemiology of bovine tuberculosis in resource-limited settings. This information is now more than ever critical for developing bovine tuberculosis control strategies such as disease free zoning and targeted animal movement restrictions in Africa as an alternative to the cost intensive “test and slaughter” with compensation that is currently practiced in high income settings. The reporting of 21 novel spoligotypes in this study indicates the overall dynamic state of bTB epidemiology in Cameroon and the need for a surveillance/control system.

## Materials and Method

### Study Design

This is a molecular and quantitative analysis of *M.bovis* isolates from our recently published field work on the burden of *Mycobacterium* in Cameroon^15^. The study was conducted in four regional abattoirs; Bamenda (North-West Region), Ngaoundere (Adamawa Region), Garoua (North Region) and Maroua (Extreme North Region) (Fig. 1).

Briefly; our sample size estimation of ~1000 cattle per regional abattoir was calculated based on a 5% lesion prevalence that had earlier been reported in the North-West Region^53^. This estimate ensured a minimum recovery of 25 isolates per abattoir with a 50% recovery rate from laboratory culture of *Mycobacterium*, in other-words ± 1.3% and 95% precision and confidence respectively. Logistical constraints limited our ability to sample extensively in Garoua and Maroua, therefore the sampling in each of these areas was limited to a week.

### Sampling Protocol

The sampling technique has been described in detail in our earlier publication^15^. Briefly, temporary reusable tags were used to identify animals and their respective plucks (respiratory tract and heart) and livers at slaughter. A blood sample was taken and the sex, number of permanent incisor teeth, body condition^54^, origin and name of the owner/butcher were recorded. The number of permanent incisor teeth was used to estimate the age.

Abattoir post mortem inspection was carried out as sanctioned by the 2002 MINEPIA inspection framework^55^. The lungs, liver, kidneys, mammary glands, retropharyngeal, submandibular, bronchial, mediastinal and mesenteric lymph nodes were examined visually and by palpation and incision for presence of granulomatous bTB-like lesions. This was done at all abattoirs by the veterinary inspectors, with the exception of Ngaoundere where the retropharyngeal and submandibular lymph nodes were examined by the research team.

A sterile blade was used between each animal sampled, while for cases where more than one sample was collected from an animal the scalpel blade and forceps were disinfected using 70% alcohol for at least 5 minute. In addition, we collected a retropharyngeal lymph node from a random sample of animals classified as non-lesioned by the meat inspectors (identified using random number generator www.Random.org). Samples from Bamenda were maintained through a cold chain using an ice park and delivered to the Tuberculosis Reference Laboratory (TBRL). Those from Ngaoundere, Garoua and Maroua were stored in a liquid nitrogen dry shipper (Taylor-Wharton) and transported to the TBRL Bamenda and stored at −80 °C until processed.

### Mycobacterial Culture

The culture method has also extensively been described in the same publication^15^. Briefly, tissue was ground to a paste in a pestle and mortar, decontaminated in 4% NaOH for 15 min followed by centrifugation at 3200 g for 20 minutes at 18 °C. The pellet was re-suspended in sterile PBS and inoculated in a Mycobacterial Growth Indicator Tubes (MGIT) using the BACTEC MGIT 960 as outlined in the manufacturer’s standard user manual, and also onto two Lowenstein Jensen (LJ) slopes (one supplemented with pyruvate and the other with glycerol). LJ cultures were examined weekly until growth was observed. At 12 weeks they were classed as “no growth observed”. Microscopic acid fast examination was done for all visible bacterial colonies to determine presence of Acid fast bodies(AFB)^56^. MGIT Tubes were monitored automatically and examined visually after 56 days. The presence of AFB was confirmed the same way as for the solid media from any suspected growth.

### Typing of acid fast bacilli (AFB)

We extracted DNA from each sample with AFB present in their colony material using the GenoLyse kit (Hain Lifescience, GmbH, Nehren, Germany) a portion of which was then typed using the Hain GenoType MTBC assay and GenoType *Mycobacterium* CM/AS kits (Hain Lifescience,GmbH, Nehren, Germany) following the manufacturer’s instructions and as previously described^57^. Species identification was done in accordance with reference to band pattern characteristics as provided by the manufacturer.

### Molecular strain typing of *M. bovis* isolates

The other portion of the extracted DNA was sent for deletion analysis, spoligotyping and MIRU-VNTR typing to Genoscreen (Lille France).

Deletion typing for the RDAf1, RDAf2 and RDEu1 deletions was determined by a PCR assay using the published primers that flank their specific defined chromosomal regions as previously described^27–29^. Isolates were termed ‘unclassified’ if they had intact PCR product for Africa1, Africa 2 and Europe 1 clonal complexes and ‘inconclusive’ or ‘mixed’ if they had both an intact and deleted outcome for any of the complexes.

Spoligotyping was carried out as previously described at Genoscreen (Lille France) using the Luminex microbeads method^58, 59^. The results were recorded as “1’ for spacers present and ‘0’ for spacers deleted. Spoligotype names were obtained from the www.mbovis.org global database by comparing the patterns to that in the database. Isolates with patterns not previously reported (novel spoligotypes) were submitted to the database for the assignment of names.

MIRU-VNTR typing used the 24 standardized loci^49^. Loci that failed to yield an amplified product or gave more than one alleles were repeated once. If it failed a second time it was recorded as a fail. If more than one product size was observed all were recorded. All the samples with failed amplified product were excluded from the analysis of the results. *M. bovis* BCG was used as the positive control while sterile molecular grade water was used as the negative control.

### Data management and statistical analysis

Field and laboratory data records were hand written and transcribed to an Access (Microsoft) database and Excel (Microsoft) spread-sheets respectively. Deletion, spoligotype and MIRU-VNTR and deletion results were merged with these data using the R statistical software tool (www.R-project.org) and written out to tables in a PostgresSQL (http://www.postgresql.org) database.

### Allelic diversity of MIRU-VTNR loci

*M. bovis* allelic diversity was estimated for each of the 24 MIRU-VNTR loci. This gives a measure of how much each locus contributes to the total discriminatory power of the MIRU-VNTR as a typing tool. The allelic diversity (h) for each locus was estimated using the following formula^50^:

1 $$h=1-\sum {x}_{i}^{2}(\frac{1}{n(n-1)})$$

where x_i_ is the frequency of the i^th^ allele at the locus and n is the number of isolates in the analysis.

### Discriminatory power calculation

The discriminatory index (D) was estimated using the Hunter and Gaston equation^60^. It is defined as the probability that the typing tool would distinguish two randomly chosen strains, sampled consecutively. The index is given by the following equation:

2 $$D=1-\frac{1}{N(N-1)}\sum _{J=1}^{S}{n}_{j}({n}_{j}-1)$$

where N is the total number of genotypes based on spoligotyping and MIRU-VNTR combined in the sample, s is the total number of unique genotypes, and n_j_ is the number of genotypes belonging to the j^th^ genotype. Since the method also inherently describes the diversity of a population from a sample it was used to calculate the genetic/molecular diversity (θ) per Region in this study.

### Phylogenetic analysis

*M. bovis* clonal complexes were assigned based on published criteria^9, 27, 28^. *M. bovis* genotypes were defined by combining spoligotype and MIRU-VNTR typing data. Genetic relationships of genotypes were deduced from distance matrices and presented by the reconstruction of an unweighted pair group average (UPGMA) tree, as well as the minimum spanning tree (MST) using the Ridom MLVA compare software (Ridom GmBH, Münster, Germany). The minimum phylogenetic distances were set at a single locus variation (SLV). Furthermore, MIRU-VNTR types were deemed homoplastic if they shared more than one different spoligotype.

An animal was classified as multiple strain infected if; two or more genotypes were isolated from the same or different anatomical compartments of the animal^46, 61^.

### Transmission estimates for *M. bovis* in Cameroon

Two transmission indices^37^ were calculated and compared for each abattoir. The recent transmission index (RTI) is an index of genetic clustering which is intended to reflect the extent of recent transmission of tuberculosis^37^. This was calculated using the formulae below;

3 $$RTI=(\frac{n-g}{n-1})$$

where n is the sample size and g is the total number of unique genotypes.

The transmission mutation index (TMI) is a measure of the mutations attributable to a transmission event. This index is calculated using the formula^37, 62^:

4 $$TMI=\tilde{\mu }(\frac{n-g+{v}_{1}}{{v}_{1}})$$

where $\tilde{\mu }$ is an independent estimate of the mutation rate of the genetic marker, g the number of unique genotypes and v_1_ is the number of single-step mutation events inferred from the data^37, 62^. We used a composite mutation rate for spoligotypes of 0.039 events per year and MIRU-VNTR mutation rate of 0.0027 per loci per year calculated from previous estimates in the literature^30, 37, 62^. Transmission or recent host contact characteristics were also evaluated by examining the molecular distances between genotypes recovered in this study. Molecular distance here is defined as the number of MIRU-VNTR loci variations between any isolates with the same Spoligotype (Figure S3).

### Cluster size characteristics

The infinite alleles model (IAM) was used to compare the observed and expected cluster size distribution. The aim here was to investigate the suitability of the IAM in explaining the observed molecular characteristics in Cameroon. The theoretical cluster size distribution conditioned on the sample size *n* of each abattoir under the IAM can be calculated using the formula below^63^

5 $$E[\alpha (j|n)]=\frac{\theta }{j}\frac{(\frac{\theta +n-j-1}{n-j})}{(\frac{\theta +n-1}{n})}$$

where θ is the diversity in that abattoir as estimated using the Hunter-Gaston equation above, n is the sample size from a given abattoir, j is the genetic cluster size and the term *E*[*α*(*j*|*n*)] is the expected frequency of the genetic cluster size. Effective population in genetics is defined as the number of individuals in a population that contribute off-springs to the next generation^31^ and Luciano argues that it’s a measure of how readily a population maintains it’s diversity^31^. In this study we have considered effective population to be a subpopulation of infected hosts carrying *M.bovis* genotypes that cause pathology and therefore could be transmitted. A χ^2^ test was used to determine if there was a significant difference between the observed and expected genetic cluster size distribution.

### Ethical statement

Although this study involved the use of biological tissue from routine post mortem inspection by veterinary public health officials in Cameroon, no experimental work was done on live vertebrates. Tissue sample collection and processing procedures were conducted according to the manual of best practice with an aim of reducing contamination. Research approval at local level was provided by the supervisory of commercial slaughterhouses sanctioned through the Ministry of Livestock, Fisheries and Animal Industries in Cameroon. The research project was also reviewed and approved based on the Animal Scientific Procedures Act of 1986 by the University of Edinburgh Ethical Review Committee (ERC No: OS02–13).

### Data Availability

The novel spoligotypes produced from this work are available in the *Mycobacterium bovis* spoligotyping database, http://www.mbovis.org/database.php (Search by country).

The datasets generated during and/or analyzed during the current study are available as a csv file in the supplementary material (SUPPLEMENTARY_MATERIAL-SREP-16-15561E.csv).

## Electronic supplementary material

Supplementary Information

Supplementary file
