# Supplementary material for: Molecular epidemiology of Mycobacterium bovis in Cameroon
Source: Sci Rep. 2017 Jul 5;7:4652. doi: 10.1038/s41598-017-04230-6 (PMC5498612; doi:10.1038/s41598-017-04230-6)
Supplement: Supplementary file 1 — Supplementary Information [file 41598_2017_4230_MOESM1_ESM.pdf]

Molecular epidemiology of *Mycobacterium bovis* in Cameroon

N. F. Egbe[1,2+], A. Muwonge[1*+], L. Ndip[3,8], R. F. Kelly[1,9], M. Sander[4], V. Tanya[5], V. Ngu Ngwa[6], I.G. Handel[1], A. Novak[1], R. Ngandalo[7] S. Mazeri[1], K. L. Morgan[10], A. Asuquo[2], B. M. de C. Bronsvoort[1, 11]

Supplementary Material

Diversity and Distribution of M. bovis spoligotypes in Central and West Africa

Af1 is the most prevalent clonal complex in West Africa with spoligotype SB0944 the most prevalent spoligotypes found in all the countries from which isolates have been reported (Figure S1 and Table S5). Furthermore, SB0944 lies at the centre of the minimum spanning tree from which most of the spoligotypes in this region are rooted (Figure S1). Although generally some countries have similar spoligotype profiles, there are subtle country-specific differences, for example between 90-95% percent of *M. bovis* spoligotypes observed in Chad, Nigeria and Cameroon belong to the Af1. On the other hand only between 60-76% of the *M. bovis* spoligotypes in Mali and Burkina Faso belong to Af1. On the other hand Spoligotypes SB1420 to SB1445 are unique to Nigeria.

SB0300 is present in Burkina Faso, Mali and Cameroon while SB1099, SB1025 and SB0951 are all present in Chad, Nigeria and Cameroon. Figures 4 and S2 also show as two clusters of spoligotypes one of which appears to be predominantly native to Cameroon and is characterized by the absence of spaces 10-17 in their pattern. (This cluster group includes spoligotypes like SB0953, SB1461, SB2161, SB2315, SB2315 and SB2321). Similarly Mali and Burkina Faso have a cluster of unique spoligotypes characterised by the absence of space 3-5.

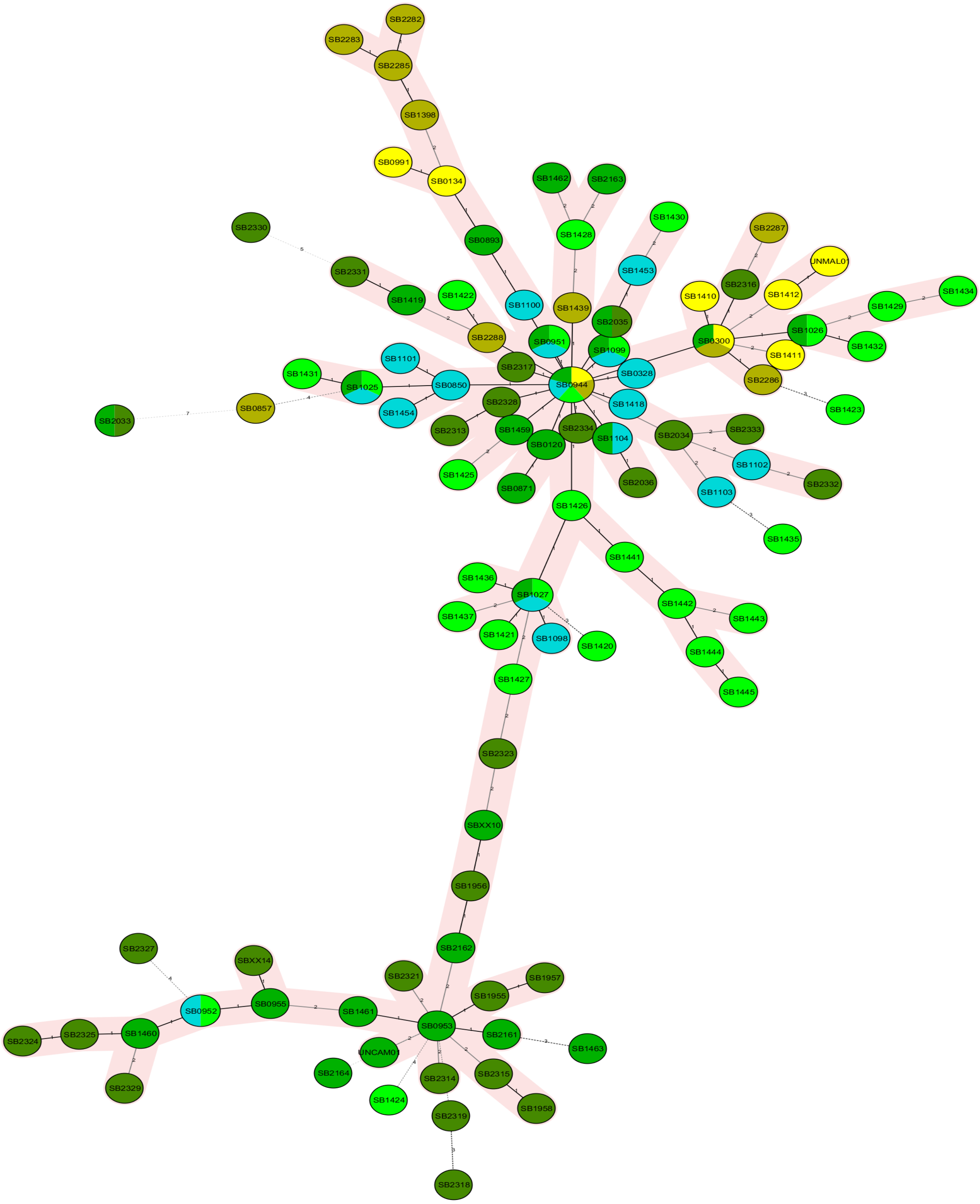

Figure S1: Minimum spanning tree of current study spoligotypes with spoligotypes reported in West and Central Africa. The dark and light green represents previously and newly reported spoligotypes in Cameroon, brown, light blue, yellow and lime green represents spoligotypes from Burkina Faso, Chad, Mali and Nigeria respectively

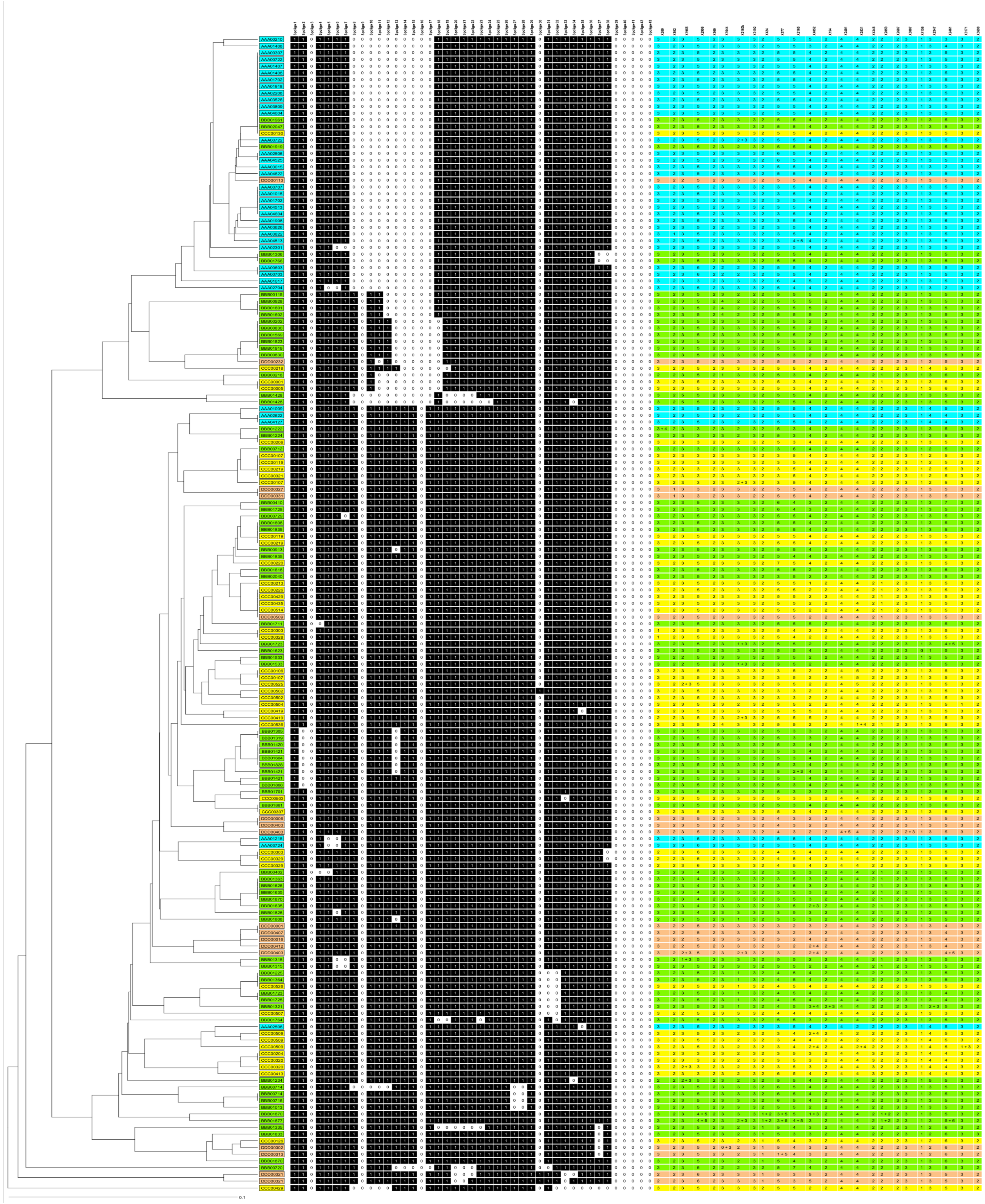

Figure S2. A UPGMA phylogenetic tree of 97 *M. bovis* “genotypes” based on the combined spoligo and MIRU-VNTR typing of cultured isolates from bTB-like lesions in cattle slaughtered at the four abattoirs sampled in Cameroon between 2012-2013. Nodes are coloured by abattoir of collection and coded with the spoligotype pattern cod, The light blue, yellow, brown and lime-green represents genotypes from Bamenda, Garoua, Maroua and Ngaoundere respectively

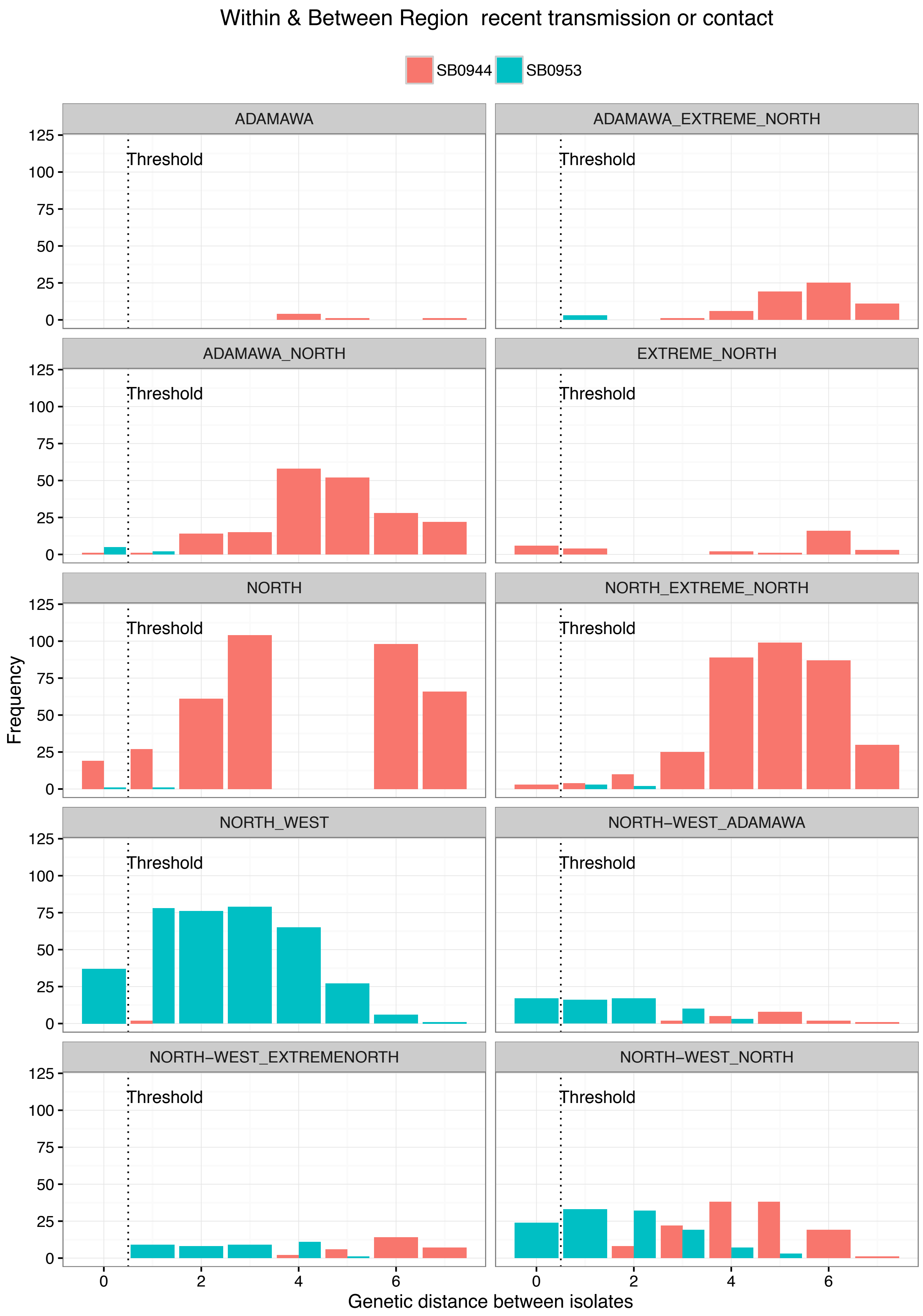

Within & Between Region recent transmission or contact
SB0944
SB0953
ADAMAWA
ADAMAWA_EXTREME_NORTH
ADAMAWA_NORTH
EXTREME_NORTH
NORTH
NORTH_EXTREME_NORTH
NORTH_WEST
NORTH−WEST_ADAMAWA
NORTH−WEST_EXTREMENORTH
NORTH−WEST_NORTH
Threshold
Frequency
125
100
75
50
25
0
0
2
4
6
Genetic distance between isolates

Figure S3: Shows the frequency of genotype pairs of the dominant spoligotypes SB0944 & SB0953 at different Genetic distances. Genetic distances 0 to 7 being the spectrum from identical to distantly related genotypes respectively. The dotted line represents the threshold below which genotypes are close enough to represent a recent contact or transmission. Genetic or molecular distance is defined as the number of MIRU-VNTR loci variations between any isolates with the same Spoligotype.

Table S1: Within Spoligotype evolution and diversity

Spoligo number	Different MIRU-VNTR types (number of types)
SB0944	V2, V5, V12, V22, V24, V25, V26, V27, V28, V32, V36, V37, V40, V43, V44, V45, V52, V53, V55, V57, V59, V60, V61, V62, V64, V65, V66, V67, V69, V70, V72, V73, V74, V75, V78 (35)
SB0953	V1, V2, V3, V4, V6, V7, V8, V9, V10, V14, V15, V17, V19, V56, V76 (15)
SB2313	V31, V39 (2)
SB1025	V38, V47, V71 (3)
SB1460	V22, V32 (2)
SB2324	V23, V58 (2)
SB0951	V21, V41, V51 (3)
SB0955	V20, V34 (2)
SB2328	V2, V49 (2)
SB1027	V29, V35 (2)
SB2035	V11, V68 (2)
SB1026	V7, V16 (2)
SB1099	V31, V54 (2)

Table S2: Attributes used for the comparative analysis between observed and IAM predicted cluster sizes

**Attributes**	**Bamenda**	**Ngaoundere**	**Garoua**	**Maroua**
Number of Isolates	37	84	42	17
Number of Unique Genotypes	20	46	26	11
Number of clusters and size	4(1,2,5,11)	5(1,2,3,5,6)	3(1,2,5)	2(1,2)
Genotypes per cluster	(15,3,1,1)	(51,8,2,2,1)	(26,6,2)	(11,3)
Observed Cluster size frequency	(15,3,1,1)	(51,8,2,2,1))	(26,6,2)	(11,3)
Predicted Cluster size frequency*	(9,4,1,0)	(9,5,3,2,2,)	(9,4,2)	(9,4)
Single locus variation genotypes	11	24	9	3

The predicted cluster size frequency under using IAM model, values are presented have been rounded of to the closest whole number. The numbers of isolates used come from the results reported in the result section, with the exception of five isolates that have been dropped from this analysis because their MIRU-VNTR loci were not complete so a genotype could not be determined. The numbers of unique genotypes were calculated by grouping all genotypes by region of origin. Cluster size, Genotype per cluster and observed frequency were determined by tabulating the frequency of each genotype in each region. The predicted cluster size frequency is calculated base on formulae 5 explained in the materials and methods section of the article. The Genotypes with a single locus variation were retrieved from the UPGMA phylogenetic tree or the Minimum spanning tree.

Table S3: Homoplasy of MIRU-VNTR in Cameroon

MIRU-VNTR	Different Spoligotypes
V2	SB0944, SB0953, SB2315, SB2317, SB2321, SB2328
V22	SB0944, SB1460, SB2033, SB2325
V7	SB0953, SB1026
V29	SB1027, SB2323
V24	SB0944, SB0893
V31	SB2313, SB1099
V32	SB0944, SB1460
V40	SB0944, SB2316
V41	SB0951, SB2330
V42	SB2318, SB2319
V45	SB0944, SB2334
V60	SB0944, SB1459
V69	SB0944, SB0120
V77	SB2332, SB2333

Table S4: The allelic diversity of the 24 MIRU-VNTR loci

MIRU-VNTR locus#	Locus Name (MIRU-VNTR Alias)	Number of alleles	Frequency of the different alleles	Allelic diversity	Conclusion
2165	ETR A	6	2 (4), 3 (46), 4 (15), 5 (157), 6 (1), 7 (1)	0.459	Moderately discriminate
2461	ETR B	7	2 (2), 3 (2), 4 (18), 5 (181), 6 (17), 7 (1), 8 (3)	0.332	Moderately discriminate
577	ETR C	5	3 (55), 4 (15), 5 (136), 6 (13), 7 (5)	0.561	Highly discriminate
580	MIRU 4; ETR D	3	1 (2), 2 (10), 3 (212)	0.098	Poorly discriminate
3192	MIRU 31; ETR E	2	2 (6), 3 (218)	0.048	Poorly discriminate
154	MIRU 2	1	2 (224)	0.004	Poorly discriminate
960	MIRU 10	1	2 (224)	0.004	Poorly discriminate
1644	MIRU 16	4	1 (1), 2 (7), 3 (213), 4 (3)	0.091	Poorly discriminate
2059	MIRU 20	2	1 (26), 2 (198)	0.202	Poorly discriminate
2531	MIRU 23	3	2 (2), 4 (217), 5 (5),	0.057	Poorly discriminate
2687	MIRU 24	2	2 (220), 3 (4)	0.031	Poorly discriminate
2996	MIRU 26	4	3 (21), 4 (12), 5 (180), 6 (11)	0.337	Moderately discriminate
3007	MIRU 27; QUB-5	2	2 (3), 3 (221)	0.022	Poorly discriminate
4348	MIRU 39	2	2 (221), 3 (3)	0.022	Poorly discriminate
802	MIRU 40	2	1 (3), 2 (221)	0.022	Poorly discriminate
424	Mtub04	2	1 (7), 2 (217)	0.056	Poorly discriminate
1955	Mtub21	3	2 (9), 3 (213), 5 (2)	0.09	Poorly discriminate
2347	Mtub29	4	1 (2), 2 (15), 3 (194), 4 (13)	0.239	Poorly discriminate
2401	Mtub30	3	2 (5), 3 (1), 4 (218)	0.048	Poorly discriminate
3171	Mtub34	2	1 (2), 3 (222)	0.013	Poorly discriminate
3690	Mtub39	2	2 (223), 3 (1)	0.004	Poorly discriminate
2163b	QUB-11b	3	1 (11), 2 (33), 3 (180)	0.327	Moderately discriminate
4052	QUB-26	5	1 (1), 2 (40), 3 (36), 4 (135), 5 (12)	0.574	Highly discriminate
4156	QUB-4156	2	0 (2), 1 (222)	0.013	Poorly discriminate

These numbers are referenced by the first four digits of their location on the *Mycobacterium tuberculosis* H37RV genome; n = 224

Table S5: Distribution of Spoligotypes in this study previously reported in Cameroon and Africa

Spoligo number	n (%)	Clonal complex in this study	Previous reported regions in Cameroon	Reported African countries
SB0944	107 (42)	Africa 1	Adamawa, Extreme North, North, East	Mali, Burkina Faso, Nigeria, Chad, Sudan, Niger
SB0953	53(20.8)	Africa 1	Adamawa, North West	None
SB1025	11(4.3)	Africa 1	Unreported	Nigeria, Chad
SB1460	9 (3.5)	Africa 1	Adamawa, Extreme North, North	None
SB0951	5 (2.0)	Africa 1	Adamawa, Extreme North, North	Nigeria, Chad
SB0955	4 (1.6)	Africa 1	Adamawa	None
SB0300	3 (1.2)	Africa 1	Unreported	Burkina Faso, Niger, Mali
SB1027	3(1.2)	Africa 1	Unreported	Nigeria, Chad
SB2035	3(1.2)	Africa 1	Cameroon	None
SB1026	2 (0.8)	Africa 1	Unreported	Nigeria
SB1099	2(0.8)	Africa 1	Unreported	Nigeria
SB1459	2(0.8)	Africa 1	Extreme North, North	None
SB2033	2(0.8)	Africa 1	Cameroon	None
SB2162	2(0.8)	Africa 1	North West	None
SB0120	1 (0.4)	Africa 1	Unreported	Algeria, Zambia, Tunisia, Ethiopia, South Africa
SB0893	1(0.4)	Africa 1	Unreported	France
SB1104	1(0.4)	Africa 1	Unreported	Chad
SB1418	1(0.4)	Africa 1	Unreported	Chad

Where n is the absolute number of spoligotype, total number of screened isolates was 255
